# Investigation of Congenital Anomalies Attributable to Known and Unknown Etiologies in a Mexican Hospital-Based Setting: Remarks on Causal Heterogeneity

**DOI:** 10.7759/cureus.88452

**Published:** 2025-07-21

**Authors:** Victor M Salinas-Torres, Rafael A Salinas-Torres, Jesus S Velarde-Felix, Jorge G Sanchez-Zazueta, Juan J Rios-Tostado, Luis A Ochoa-Ramirez

**Affiliations:** 1 Department of Genomic Medicine, Servicios de Salud del Instituto Mexicano del Seguro Social para el Bienestar, Hospital General Culiacán, Culiacán, MEX; 2 Department of Human Genetics and Genomics, Servicios de Salud del Instituto Mexicano del Seguro Social para el Bienestar, Hospital General Durango, Durango, MEX; 3 Faculty of Medicine, Universidad Autónoma de Sinaloa, Culiacán, MEX; 4 Department of Systems and Computing, Instituto Tecnológico de Tijuana, Tijuana, MEX; 5 Faculty of Biology, Universidad Autónoma de Sinaloa, Culiacán, MEX

**Keywords:** causality, congenital anomalies, etiology, mexico, pregnancy outcome

## Abstract

Background

Congenital anomalies (CA) are a major contributor to infant mortality worldwide, and the risk of CA differs by maternal and fetal characteristics. Moreover, studies establishing the etiology of CA are methodologically heterogeneous, and its investigation among the Hispanic population is limited. This study aims to investigate the potential etiologies for CA and compare their etiologic profile across pregnancy outcome, fetal/infant sex, and maternal age in a Mexican population-based setting.

Methods

Potential causes of CA from 2022 to 2024 at Durango General Hospital (northwest Mexico) were investigated through a hospital-based CA surveillance program. Prevalence and 95% confidence intervals (CI) were calculated for known and unknown etiologies and clinical features (live births, fetal losses, males, females, and maternal age of <20, 20-34, and ≥35 years). Pearson’s chi-squared test and Fisher’s exact test were used to analyze between-group diﬀerences. Two-tailed probability values of <0.05 were considered statistically significant.

Results

Altogether, 497 cases among 11608 births (prevalence, 4.2%; 95% CI, 3.9%-4.6%) were considered. In 155 cases (31.1%, 95% CI: 27.2%-35.3%), a definitive cause was assigned. Pathogenic processes were recognized in 53 cases (10.6%, 95% CI: 8.2%-13.6%) among 342 cases of unknown etiology. Fetal losses, males, and maternal age of <20 years were more prevalent among CA compared to the underlying birth cohort (all P< 0.05). Among recognized etiologies, there were significant differences between fetal losses and pathogenesis classification; males and the environmental etiology group; maternal age of <20 years and the genetic etiology group; single/multiple gene disorders, family history, and multiple exposures; and maternal age of ≥35 years and chromosomal abnormalities, the environmental etiology group, maternal illness, and pathogenesis classification (all P< 0.05). Among unknown etiologies, significant differences were observed in fetal losses and other/multiple categories, live births and craniofacial and musculoskeletal defects, males and cardiovascular and genitourinary defects, females and the unknown etiology group and craniofacial and musculoskeletal defects, maternal age of <20 years and abdominal defects, and maternal age of 20-34 years and the unknown etiology group and craniofacial defects (all P < 0.05).

Conclusions

The recognized potential nature for CA in this cohort was 41.8%. This study identified causal heterogeneity and provided practical value among its associated clinical features. It emphasizes the relevance of thoughtful clinical investigation into CA despite the predominant unknown causality.

## Introduction

Congenital anomalies (CA) include any structural or functional anomaly likely to result from genetic and environmental causes affecting about 5% of live births, despite sample sizes, methodologies, or geographical settings [1]. However, estimations on CA may account for more than 10% of miscarriages, outnumbering infant deaths [2]. Notably, these outcomes are particularly high in the Hispanic population, which poses a considerable interracial variation for CA, in addition to the burden of mortality among low-income countries [2,3]. Moreover, the risk of CA differs by maternal and fetal characteristics, raising questions about additional insight into its causes.

Indeed, understanding the environmental and genetic influences predisposing to CA can be used to design preventive public health strategies, whereas accurate diagnoses also contribute to specific prognosis and genetic counseling for affected individuals and their families [4]. Notwithstanding the progress in elucidating the etiology of human CA, established causality from previous population-based studies varies between 8.8% and 78.7% [5-10]. The main reasons behind this wide variation include sources of CA ascertainment, variability around eligibility criteria related to the presentation of CA, and methodological heterogeneity, thus highlighting the need for comprehensive research on the etiology of CA and its associated clinical features.

To further analyze the causes of CA, in a Mexican hospital-based surveillance program among 11608 births from northwest Mexico, this study aimed to investigate the potential etiologies for 497 infants with CA and compare their etiologic profile and clinical features of CA across groups stratified by pregnancy outcome, fetal/infant sex, and maternal age.

## Materials and methods

Study population and ethics statement

From July 2022 to May 2024, this study assessed CA among all pregnancy outcomes (live births, stillbirths, and pregnancy terminations) at Durango General Hospital. The latter is located in the northwestern region of Mexico (Durango state). It is the regional center for specialized maternal and child care of 39 municipalities with a population of nearly two million inhabitants. The study was approved by the Ethics Committee of the Durango Secretary of Health (approval number: 007/2023) and followed the Strengthening the Reporting of Observational Studies in Epidemiology (STROBE) guidelines.

Data source, quality control, and eligibility criteria

The data source for this study was the hospital-based CA surveillance program, which employed detailed clinical information, as well as appropriate imaging and laboratory results for each case, based on both prenatal and postnatal clinical records. The investigations were performed according to the needs of each case, with the assistance of an expert multidisciplinary team (hospital staff). Data were collected by experienced doctors using a standardized form, and the principal investigator supervised the process daily to conduct comprehensive quality control. All fetal/infant cases were identified as those with at least one major CA and were evaluated by a clinical geneticist, who performed a detailed dysmorphology examination and also interviewed at least one parent to collect additional background data (previous obstetrics history and family histories). Cases without a major CA were included if they had specific laboratory results (e.g., trisomy 21). Exclusion criteria included spontaneous abortions and pregnancy terminations occurring at <20 weeks of gestation.

Etiologic classification

CA potential etiologic profiles were classified as known or unknown causes and were tabulated considering the following features: pregnancy outcome (live births and fetal losses {the latter included stillbirths and pregnancy terminations due to their small number of cases}), fetal/infant sex (male and female), and maternal age (grouped in <20, 20-34, and ≥35 years).

Initially, all CA were classified according to the World Health Organization International Classification of Diseases (10th revision) and grouped by anatomical system defects: craniofacial, cardiovascular, digestive, genitourinary, musculoskeletal, abdominal, other/multiple, and chromosomal abnormalities [11]. Subsequently, possible diagnoses and causes of known etiology were assigned based on established criteria and were categorized into genetic, environmental, gene-environment interactions, and twinning [12].

Genetic cases were classified as chromosomal abnormalities (number and structure, through karyotype analysis), single/multiple gene presumed abnormalities (based on clinical criteria [12] or when molecular testing was available), and family history of CA and consanguinity (first, second, third, or distant degree). Environmental etiology included clinical background and self-reported exposure to a recognized human teratogen [13]. The latter was classified as maternal medications/drugs (e.g., anticonvulsants/cocaine), maternal illness (e.g., pregestational/gestational diabetes [14,15]), intrauterine infections (e.g., TORCH complex [16]), mechanical forces (e.g., amnion rupture), occupational exposure (e.g., agricultural pesticides), or multiple exposures (inferred from two or more self-reported recognized human teratogens). Gene-environment causality was collected from self-reported complex interactions of the above influences (e.g., family history of CA in association with environmental exposure). Twinning etiology included any twin-associated major anomaly (e.g., conjoined twins).

Regarding CA of unknown etiology, further classification based on embryonic recognized pathogenic processes was employed to assign a potential pathogenesis (developmental field defect, known pattern {association, complex, or spectrum}, and sequence) [12,13].

Statistical analysis

Statistical analyses were performed employing the SPSS software version 21 (IBM Corp., Armonk, NY). Data were expressed as numbers (n) and percentages (%). The CA potential etiologic profile was presented as case counts by category and subtype. Crude prevalence was calculated as the quotient of the total number of CA and the total number of pregnancy outcomes. Stratified prevalence and 95% confidence intervals (CI) were calculated for each category and subtype. Pearson’s chi-squared test and Fisher’s exact test were used to compare the distribution of CA among live births and fetal loss case counts, as well as the between-group diﬀerences among fetal/infant sex and maternal age. Two-tailed probability values of <0.05 were considered statistically significant.

## Results

Known and unknown causes of CA

**Figure 1:**
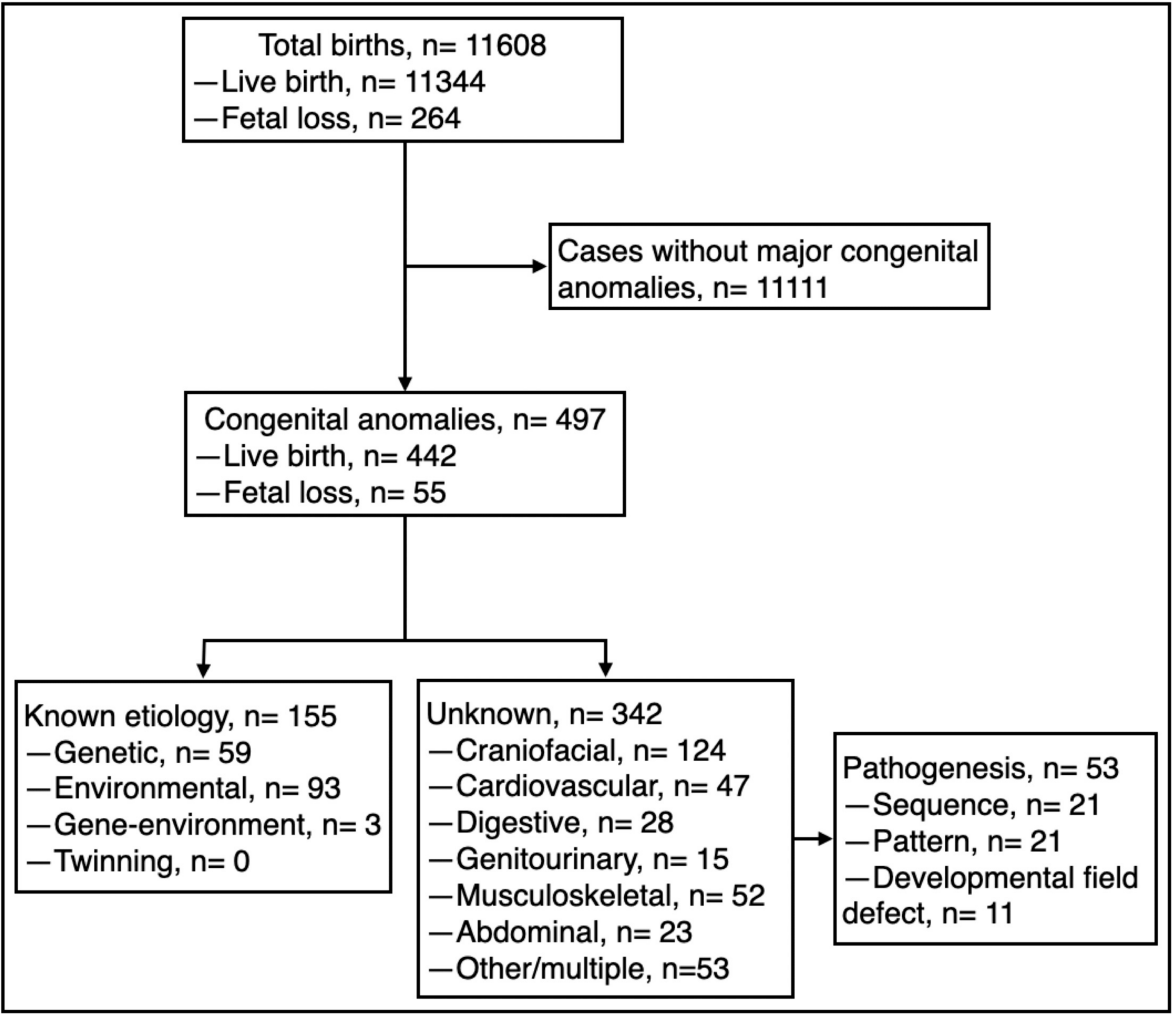
Flowchart of total births and potential etiologic profile of 497 cases with congenital anomalies

This study considered 497 infants with CA among 11608 total births for a prevalence of 4.2% (95% CI: 3.9%-4.6%). Altogether, 155 CA (31.1%, 95% CI: 27.2%-35.3%) were assigned to a known etiology, and 342 CA (68.8%, 95% CI: 64.6%-72.7%) were classified as unknown etiology; among the latter group, 53 cases (10.6%, 95% CI: 8.2%-13.6%) were further classified into a recognized pathogenesis (Figure 1). There were no twin-associated major anomalies in either the 36 sets of twins or the two sets of triplets.

Etiologic classification of CA stratified by pregnancy outcome

Overall, there were 11344 live births and 264 fetal losses, including 442 CA (3.9%, 95% CI: 3.5%-4.2%) and 55 CA (20.8%, 95% CI: 16.3%-26.1%), respectively, showing statistically significant differences between the groups (P < 0.001).

Fetal losses were over-represented in both other/multiple categories (30.9%, P < 0.001) and pathogenesis classification (23.6%, P = 0.003); meanwhile, live births were more prevalent in craniofacial (27.1%, P < 0.001) and musculoskeletal (11.5%, P = 0.019) anatomical system defects. No statistically significant differences were found among genetic and environmental categories and other etiologic profiles (Table 1).

**Table 1:** Etiologic classification and prevalence rates of congenital anomalies stratified by pregnancy outcome ^a^Pearson’s chi-squared test ^b^Fisher’s exact test ^†^Pathogenesis classification included grouped cases of developmental field defect, known pattern, and sequence, which were assigned from cases within the unknown etiology group CI: confidence interval

Etiology	Congenital anomalies				Chi-squared value	P value
	Live birth, n = 442		Fetal loss, n = 55			
	n	Prevalence (95% CI)	n	Prevalence (95% CI)		
Genetic	52	11.7 (9.0-15.1)	7	12.7 (6.3-24.0)	0.043	0.825^a^
Environmental	80	18.1 (14.7-21.9)	13	23.6 (14.3-36.3)	0.985	0.320^a^
Gene-environment	2	0.4 (0.1-1.6)	1	1.8 (0.3-9.6)	1.520	0.297^b^
Unknown	308	69.6 (65.2-73.7)	34	61.8 (48.6-73.4)	1.409	0.235^a^
Craniofacial	120	27.1 (23.2-31.4)	4	7.2 (2.8-17.2)	10.32	<0.001^b^
Cardiovascular	44	9.9 (7.5-13.1)	3	5.4 (1.8-14.8)	1.156	0.460^b^
Digestive	24	5.4 (3.6-7.9)	4	7.2 (2.8-17.2)	0.312	0.535^b^
Genitourinary	13	2.9 (1.7-4.9)	2	3.6 (1.0-12.3)	0.080	0.676^b^
Musculoskeletal	51	11.5 (8.8-14.8)	1	1.8 (0.3-9.6)	4.933	0.019^b^
Abdominal	20	4.5 (2.9-6.8)	3	5.4 (1.8-14.8)	0.095	0.731^b^
Other/multiple	36	8.1 (5.9-11.0)	17	30.9 (20.2-44.0)	26.60	<0.001^b^
Pathogenesis^†^	40	9.0 (6.7-12.0)	13	23.6 (14.3-36.3)	10.92	0.003^b^

Etiologic classification of CA stratified by fetal/infant sex

Fetal/infant sex assignment between the underlying birth cohort constituted 5933 males and 5671 females, and four cases were affected with ambiguous genitalia, in which it was not possible to determine the sex. Compared with the affected cohort, including 282 males (4.7%, 95% CI: 4.2%-5.3%) and 211 females (3.7%, 95% CI: 3.2%-4.2%), statistically significant differences between the groups were found (P = 0.004). 

Males were documented more frequently in the environmental etiology group (21.9%, P = 0.040) and cardiovascular and genitourinary anatomical system defects (12.0%, P = 0.030, and 3.9%, P = 0.047, respectively). Conversely, females were more common in the unknown etiology group (75.8%, P = 0.002) and craniofacial and musculoskeletal anatomical system defects (34.1%, P < 0.001, and 15.1%, P = 0.004, respectively). No statistically significant differences were found among genetic and environmental categories and other etiologic profiles (Table 2).

**Table 2:** Etiologic classification and prevalence rates of congenital anomalies stratified by fetal/infant sex ^a^Pearson’s chi-squared test ^b^Fisher’s exact test ^*^Frequencies may not add to the total due to indeterminate sex ^†^Pathogenesis classification included grouped cases of developmental field defect, known pattern, and sequence, which were assigned from cases within the unknown etiology group CI: confidence interval

Etiology	Congenital anomalies				Chi-squared value	P value
	Male, n = 282		Female, n = 211			
	n	Prevalence (95% CI)	n	Prevalence (95% CI)		
Genetic	40	14.1 (10.5-18.7)	19	9.0 (5.8-13.6)	3.073	0.092^a^
Environmental	62	21.9 (17.5-27.1)	31	14.6 (10.5-20.1)	4.195	0.040^a^
Gene-environment	2	0.7 (0.1-2.5)	1	0.4 (0.08-2.6)	0.110	0.739^b^
Unknown	178	63.1 (57.3-68.5)	160	75.8 (69.6-81.1)	9.043	0.002^a^
Craniofacial	52	18.4 (14.3-23.3)	72	34.1 (28.0-40.7)	15.76	<0.001^a^
Cardiovascular	34	12.0 (8.7-16.3)	13	6.1 (3.6-10.2)	4.864	0.030^b^
Digestive	17	6.0 (3.8-9.4)	11	5.2 (2.9-9.0)	0.149	0.844^b^
Genitourinary^*^	11	3.9 (2.1-6.8)	2	0.9 (0.2-3.3)	4.099	0.047^b^
Musculoskeletal	20	7.0 (4.6-10.7)	32	15.1 (10.9-20.6)	8.338	0.004^b^
Abdominal	11	3.9 (2.1-6.8)	12	5.6 (3.2-9.6)	0.866	0.392^b^
Other/multiple^*^	33	11.7 (8.4-15.9)	18	8.5 (5.4-13.0)	1.308	0.296^b^
Pathogenesis^†^	32	11.3 (8.1-15.5)	21	9.9 (6.6-14.7)	0.244	0.661^b^

Etiologic classification of CA stratified by maternal age

Maternal age distribution among the underlying birth cohort comprised 1417 mothers of <20 years, 8320 mothers between 20 and 34 years, and 1871 mothers of ≥35 years, whereas the affected cohort included 102 (7.2%, 95% CI: 5.9%-8.6%), 281 (3.3%, 95% CI: 3.0%-3.7%), and 114 (6.0%, 95% CI: 5.1%-7.2%), respectively, showing statistically significant differences between the groups (P < 0.001).

Maternal age of <20 years was higher in the following categories: genetic (24.5%, P < 0.001), single/multiple gene disorders (7.8%, P = 0.021), family history (4.9%, P = 0.003), multiple exposures (3.9%, P = 0.017), and abdominal defects (9.8%, P = 0.013). Maternal age of ≥35 years was over-represented in chromosomal abnormalities (13.1%, P < 0.001), the environmental etiology group (28.9%, P = 0.005), maternal illness (25.4%, P < 0.001), and pathogenesis classification (21.9%, P < 0.001). Finally, maternal age of 20-34 years was more common in the unknown etiology group (79.3%, P < 0.001) and craniofacial defects (31.3%, P < 0.001). No statistically significant differences were observed in other categories (Table 3).

**Table 3:** Etiologic classification and prevalence rates of congenital anomalies stratified by maternal age -: Quantity zero ^a^Pearson’s chi-squared test ^†^Pathogenesis classification included grouped cases of developmental field defect, known pattern, and sequence, which were assigned from cases within the unknown etiology group CI: confidence interval

Etiology	Congenital anomalies						Chi-squared value	P value
	<20 years, n = 102		20-34 years, n = 281		≥35 years, n = 114			
	n	Prevalence (95% CI)	n	Prevalence (95% CI)	n	Prevalence (95% CI)		
Genetic	25	24.5 (17.1-33.6)	15	5.3 (3.2-8.6)	19	16.6 (10.9-24.5)	29.54	<0.001^a^
Chromosomal abnormalities	9	8.8 (4.7-15.9)	7	2.4 (1.2-5.0)	15	13.1 (8.1-20.5)	17.24	<0.001^a^
Single/multiple genes	8	7.8 (4.0-14.7)	6	2.1 (0.9-4.5)	3	2.6 (0.9-7.4)	7.659	0.021^a^
Family history	5	4.9 (2.1-10.9)	1	0.3 (0.06-1.9)	1	0.8 (0.1-4.8)	11.43	0.003^a^
Consanguinity	3	2.9 (1.0-8.2)	1	0.3 (0.06-1.9)	-	-	-	-
Environmental	18	17.6 (11.4-26.1)	42	14.9 (11.2-19.5)	33	28.9 (21.4-37.8)	10.54	0.005^a^
Maternal medications/drugs	3	2.9 (1.0-8.2)	2	0.7 (0.2-2.5)	1	0.8 (0.1-4.8)	3.253	0.196^a^
Maternal illness	6	5.8 (2.7-12.2)	34	12.1 (8.7-16.4)	29	25.4 (18.3-34.1)	18.94	<0.001^a^
Intrauterine infections	3	2.9 (1.0-8.2)	2	0.7 (0.2-2.5)	-	-	-	-
Mechanical forces	-	-	2	0.7 (0.2-2.5)	1	0.8 (0.1-4.8)	-	-
Occupational exposure	2	1.9 (0.05-6.8)	1	0.3 (0.06-1.9)	1	0.8 (0.1-4.8)	2.424	0.297^a^
Multiple exposures	4	3.9 (1.5-9.6)	1	0.3 (0.06-1.9)	1	0.8 (0.1-4.8)	8.112	0.017^a^
Gene-environment	1	0.9 (0.01-5.3)	1	0.3 (0.06-1.9)	1	0.8 (0.1-4.8)	0.671	0.714^a^
Unknown	58	56.8 (47.1-66.0)	223	79.3 (74.2-83.6)	61	53.5 (44.3-62.4)	33.79	<0.001^a^
Craniofacial	21	20.5 (13.8-29.4)	88	31.3 (26.1-36.9)	15	13.1 (8.1-20.5)	15.58	<0.001^a^
Cardiovascular	5	4.9 (2.1-10.9)	33	11.7 (8.4-16.0)	9	7.8 (4.2-14.3)	4.512	0.104^a^
Digestive	3	2.9 (1.0-8.2)	14	4.9 (2.9-8.1)	11	9.6 (5.4-16.4)	5.072	0.079^a^
Genitourinary	2	1.9 (0.05-6.8)	12	4.2 (2.4-7.3)	1	0.8 (0.1-4.8)	3.680	0.158^a^
Musculoskeletal	5	4.9 (2.1-10.9)	34	12.1 (8.7-16.4)	13	11.4 (6.7-18.5)	4.278	0.117^a^
Abdominal	10	9.8 (5.4-17.1)	11	3.9 (2.2-6.8)	2	1.7 (0.4-6.1)	8.648	0.013^a^
Other/multiple	12	11.7 (6.8-19.4)	31	11.0 (7.8-15.2)	10	8.7 (4.8-15.4)	0.598	0.741^a^
Pathogenesis^†^	11	10.7 (6.1-18.2)	17	6.0 (3.8-9.4)	25	21.9 (15.3-30.3)	21.46	<0.001^a^

Detailed potential known and unknown causes of the 497 CA are available in the Appendices.

## Discussion

The overall recognized potential nature for CA in this cohort was 41.8%, with a 95% CI of 37.5%-46.2%. Conclusive evidence for genetic and environmental factors, complex gene-environment interactions, and pathogenic processes was possible by means of clinical, imaging, and laboratory examinations. Moreover, in the analyses, higher frequencies of fetal losses, males, and maternal age of <20 years between known and unknown causes of CA were statistically significant variables, even after adjusting for other characteristics such as live births, females, and maternal age of 20-34 and ≥35 years. These results stress the heterogeneity in the etiologies of CA and novel features converging with the causes of the affected cohort.

On the one hand, the prevalence of potential causes for CA in this study diverges from previous population-based studies; while it was close to the estimates in one study [9], it varies widely among others [5-8,10]. On the other hand, the assignment of these causes and their oscillation among the above studies can vary due to several components such as sources of CA ascertainment, sample sizes, eligibility criteria related to the presentation of CA, methodological heterogeneity, sociocultural and geographical settings, and ethnic background of each population.

Furthermore, estimates of causal factors also seem heterogeneous, which might be the result of the same methodological heterogeneity mentioned above. Altogether, considering this study and previous population-based studies (n = 15357), the overall prevalence include genetic etiology, 18.2% (n = 2800, 95% CI: 17.6%-18.8%) [5-10]; environmental factors, 4.2% (n = 649, 95% CI: 3.9%-4.5%) [7-10]; complex gene-environment interaction, 10.4% (n = 1598, 95% CI: 9.9%-10.9%) [6,7,9,10]; and twinning, 0.3% (n = 53, 95% CI: 0.2%-0.4%) [5,7-10]. Therefore, the apparently identified causes for CA among population-based studies comprise one in three cases. So, the assigned known etiology in this study agrees with the latter finding.

Results from statistical analyses indicated several influential findings regarding the causality of CA. When considering pregnancy outcome, the prevalence of fetal losses (11.0%) owing to CA within this Hispanic population is higher than in other populations [7,8,10,17]. Moreover, the seemingly intrinsic relationship between fetal losses and other/multiple and pathogenesis categories, as well as live births and craniofacial and musculoskeletal defects (P < 0.05, Table 1), provides novel insight into the unknown causes of CA. Future research should explore risk factors for fetal losses, as compared with live births, to reduce interracial disparities.

Sex differences in the prevalence of human CA are common, and previous studies demonstrate that males are at greater risk for CA than females; such observation stands strengthened through opposite-sex twins’ assessments [18]. Yet, a comprehensive analysis of sex differences in a Hispanic population with a wide range of CA, comparing its etiologic profile, has not been reported. This study observed significant associations between males and the environmental etiology group, cardiovascular and genitourinary defects, and females and the unknown etiology group and craniofacial and musculoskeletal defects (P < 0.05, Table 2). Additional work examining the explanatory role of these influences would be a valuable contribution to the field, as several environmental factors, such as pesticides or toxins, may lead to CA and the discrepancy of the fetal/infant sex ratio, as it was observed in the affected cohort.

Contemporary studies on the effect of maternal age on CA have found consistent findings and have been well-recognized for both chromosomal and nonchromosomal anomalies [19]. This study identified a steady association linking this issue among abdominal and craniofacial defects, genetic etiology, single/multiple gene disorders, family history, and chromosomal abnormalities [19-21]. However, further associations were observed between mothers of <20 years and multiple exposures; mothers of ≥35 years and the environmental etiology group, maternal illness, and pathogenesis classification; and mothers of 20-34 years and the unknown etiology group (P < 0.05, Table 3). These observations may be useful as hypotheses and highlight a sizable opportunity to narrow gaps in susceptibility across maternal age and adverse pregnancy outcomes.

Evidence for an underlying pathogenic process was noted in 10.6% of cases among the affected cohort. Although the overall recognized pathogenesis estimate is higher than in other studies, some similarities according to their morphologic type have been observed [8,17]. First, the presentation of most CA was as an isolated defect (morphologic anomalies resulting from an intrinsically abnormal developmental process). Second, recognized pathogeneses of these structural defects were commonly presented as sequences. Third, in cases with multiple CA, a known pattern (association, complex, or spectrum) was more likely to be reported. Compared to known causes, these remarks can be particularly insightful for the unknown etiology cases to better inform clinical practice.

In interpreting the study’s findings, the influence of sociocultural and health system factors should be considered. Despite the importance of CA as a public health issue [1,3], there is an underrepresentation of detailed CA investigation among the Hispanic race, along with fetal cause of death prevention strategies and public awareness [2]. Moreover, variability and resource settings between facilities or surveillance programs point to areas where research could be improved, particularly among mothers of <20 and ≥35 years [5,7-10]. Some related factors may be attributed to limited access to prenatal or medical services, inadequate nutrition, or even a lack of overall health awareness [19-21]. Specific factors addressed in this study reinforce the importance and target some discussed issues. These results may be helpful to improve women’s awareness of CA, promote developmental health, and provide more adequate medical care for pregnant women.

Causes of CA in this study are limited for a number of reasons. Although it was possible to delineate cases with chromosomal aberrations by karyotype and genetic abnormalities by clinical criteria and molecular analysis (e.g., neurofibromatosis type 1 {NF1}, Werdnig-Hoffmann, and mucopolysaccharidosis), due to the resource-limited setting, there was a lack of mutation analysis in several cases [12]. Thus, an enhanced quality and specificity of the diagnostic findings through whole genome sequencing or chromosome microarray were not possible [22]. New insights from genomic tools suggest prominent pathways for future research and will add new findings to the recognized causes in infants with CA. Some maternal, placental, and fetal factors may be sensible targets from insult during the third to the eighth week of embryogenesis, prompting CA [23]. Many pregnancies were not even recognized at these early stages, and ascribing exposures was challenging, particularly considering the potential for recall bias. Diagnostic assessment for some cases among stillbirths and pregnancy terminations was limited to the review of autopsy findings; consequently, bias might be introduced in such diagnoses [24]. The above limitations were minimized by the fact that all potential cases were referred for genetic counseling, recording a sensitive and individualized collection of information through conservative established criteria and thoughtful clinical examination.

Strengths of this study include the ability to identify each affected fetus/infant with CA from daily hospital-based surveillance, including several rare defects for timely documentation [25]. The assessments of the many common and uncommon CA also made it possible to determine the etiologic heterogeneity of this affected cohort. Likewise, comprehensive research on the etiology of CA tailored to the associated pregnancy outcomes, fetal/infant sex, and maternal age allowed to identify the novel and meaningful understanding of CA attributable to known and unknown causes. This knowledge may be employed for better primary prevention interventions.

## Conclusions

Investigation in this affected cohort provides evidence that CA attributable to known causes was present in one of three cases. The identified causal heterogeneity, along with its associated clinical features, has practical value, as further examination in other population-based settings should expect to identify variation within the etiologies and elude etiologic under-ascertainment. To avoid the latter, this study emphasizes the relevance of a careful family and clinical investigation on CA, in spite of the predominant unknown causality.

The presence of specific clinical, genetic, environmental, and pathogenic findings points to a complex interplay of causal factors within this Hispanic population. Further investigation will be necessary to confirm these findings by exploring these disparities to allow for comparison in different populations.

## Appendices

Table 4 shows the detailed potential known and unknown causes of the 497 CA.

**Table 4:** Etiologic profile of 497 cases with congenital anomalies at Durango General Hospital from July 2022 to May 2024 -: Quantity zero ^ƒ^Contemplating those cases of Noonan syndrome and neurofibromatosis type 1 who also informed similarly first-degree affected relatives, family history was noted in 2.4% (n = 12) ^†^Developmental field defect ^‡^Sequence ^§^Known pattern (association, complex, or spectrum) ^‡a^Six cases with a background of fetal immobilization during late gestation from oligohydramnios OMIM, Online Mendelian Inheritance in Man; CAH, congenital adrenal hyperplasia; VATER/VACTERL, vertebral anomalies, anal atresia, cardiovascular, tracheoesophageal fistula, esophageal atresia, radial or renal, and limb; OAVS, oculoauriculovertebral spectrum; MURCS, Müllerian duct aplasia, unilateral renal agenesis, and cervicothoracic somite anomalies; OEIS, omphalocele, exstrophy of the cloaca, imperforate anus, and spinal defects; KTW, Klippel-Trenaunay-Weber; CMN, Casamassima-Morton-Nance

Etiology	Congenital anomalies, n (%)		
	Live birth, n = 442	Fetal loss, n = 55	Total, n = 497
Known	134 (30.3)	21 (38.1)	155 (31.1)
Genetic	52 (11.7)	7 (12.7)	59 (11.8)
Chromosomal abnormalities	26 (5.8)	5 (9.0)	31 (6.2)
Down syndrome	14 (3.1)	1 (1.8)	15 (3.0)
Turner syndrome	3 (0.6)	2 (3.6)	5 (1.0)
22q11.2 deletion syndrome	3 (0.6)	-	3 (0.6)
Edwards syndrome	1 (0.2)	1 (1.8)	2 (0.4)
Patau syndrome	1 (0.2)	1 (1.8)	2 (0.4)
Cri du chat syndrome	1 (0.2)	-	1 (0.2)
Wolf-Hirschhorn syndrome	1 (0.2)	-	1 (0.2)
Williams-Beuren syndrome	1 (0.2)	-	1 (0.2)
Partial trisomy 10q	1 (0.2)	-	1 (0.2)
Single/multiple genes	16 (3.6)	1 (1.8)	17 (3.4)
Noonan syndrome (OMIM 163950)	3 (0.6)	-	3 (0.6)
Neurofibromatosis type 1 (OMIM 162200)	2 (0.4)	-	2 (0.4)
Achondroplasia (OMIM 100800)	1 (0.2)	1 (1.8)	2 (0.4)
Spinal muscular atrophy type 1 (OMIM 253300)	1 (0.2)	-	1 (0.2)
Smith-Lemli-Opitz syndrome (OMIM 270400)	1 (0.2)	-	1 (0.2)
CAH 17-alpha-hydroxylase deficiency (OMIM 202110)	1 (0.2)	-	1 (0.2)
Freeman-Sheldon syndrome (OMIM 193700)	1 (0.2)	-	1 (0.2)
Aarskog-Scott syndrome (OMIM 305400)	1 (0.2)	-	1 (0.2)
Waardenburg syndrome type 1 (OMIM 193500)	1 (0.2)	-	1 (0.2)
Townes-Brocks syndrome (OMIM 107480)	1 (0.2)	-	1 (0.2)
Shprintzen-Goldberg syndrome (OMIM 182212)	1 (0.2)	-	1 (0.2)
Hurler syndrome (OMIM 607014)	1 (0.2)	-	1 (0.2)
Mucopolysaccharidosis type 2 (OMIM 309900)	1 (0.2)	-	1 (0.2)
Family history^ƒ^	7 (1.5)	-	7 (1.4)
First-degree affected relatives	5 (1.1)	-	5 (1.0)
Cleft lip/palate	2 (0.4)	-	2 (0.4)
Patent ductus arteriosus	1 (0.2)	-	1 (0.2)
Esophageal atresia with tracheoesophageal fistula	1 (0.2)	-	1 (0.2)
Piebaldism	1 (0.2)	-	1 (0.2)
Second-degree affected relatives	2 (0.4)	-	2 (0.4)
Polydactyly	1 (0.2)	-	1 (0.2)
Hypospadias	1 (0.2)	-	1 (0.2)
Consanguinity	3 (0.6)	1 (1.8)	4 (0.8)
First degree	-	-	-
Second degree	3 (0.6)	1 (1.8)	4 (0.8)
Atrioventricular septal defect	1 (0.2)	-	1 (0.2)
Cystic fibrosis	1 (0.2)	-	1 (0.2)
Phenylketonuria	1 (0.2)	-	1 (0.2)
Meckel-Gruber syndrome	-	1 (1.8)	1 (0.2)
Environmental	80 (18.0)	13 (23.6)	93 (18.7)
Maternal medications/drugs	4 (0.9)	2 (3.6)	6 (1.2)
Cigarette smoking	2 (0.4)	1 (1.8)	3 (0.6)
Alcohol	1 (0.2)	-	1 (0.2)
Valproate	1 (0.2)	-	1 (0.2)
Isotretinoin	-	1 (1.8)	1 (0.2)
Maternal illness	63 (14.9)	6 (10.9)	69 (13.8)
Diabetes	62 (14.0)	6 (10.9)	68 (13.6)
Hypothyroidism	1 (0.2)	-	1 (0.2)
Intrauterine infections	4 (0.9)	1 (1.8)	5 (1.0)
Rubella	2 (0.4)	1 (1.8)	3 (0.6)
Cytomegalovirus	1 (0.2)	-	1 (0.2)
Syphilis	1 (0.2)	-	1 (0.2)
Mechanical forces	2 (0.4)	1 (1.8)	3 (0.6)
Constricting bands	2 (0.4)	1 (1.8)	3 (0.6)
Occupational exposure	3 (0.6)	1 (1.8)	4 (0.8)
Agricultural pesticides	3 (0.6)	1 (1.8)	4 (0.8)
Multiple exposures	4 (0.9)	2 (3.6)	6 (1.2)
Gene-environment	2 (0.4)	1 (1.8)	3 (0.6)
Hypospadias	1 (0.2)	-	1 (0.2)
Ventricular septal defect	1 (0.2)	-	1 (0.2)
Cleft lip/palate	-	1 (0.2)	1 (0.2)
Twinning	-	-	-
Unknown	308 (69.6)	34 (61.8)	342 (68.8)
Craniofacial	120 (28.4)	4 (7.2)	124 (24.9)
Spina bifida	43 (9.7)	-	43 (8.6)
Hydrocephalus	33 (7.4)	-	33 (6.6)
Cleft lip/palate	26 (5.8)	-	26 (5.2)
Anotia/microtia	8 (1.8)	-	8 (1.6)
Anencephaly	5 (1.1)	2 (3.6)	7 (1.4)
Encephalocele	4 (0.9)	1 (1.8)	5 (1.0)
Microphthalmos	1 (0.2)	-	1 (0.2)
Holoprosencephaly^†^	-	1 (1.8)	1 (0.2)
Cardiovascular	44 (9.9)	3 (5.4)	47 (9.4)
Pulmonary valve atresia	9 (2.0)	-	9 (1.8)
Atrial septal defect	9 (2.0)	-	9 (1.8)
Ventricular septal defect	6 (1.3)	-	6 (1.2)
Tetralogy of Fallot^†^	4 (0.9)	1 (1.8)	5 (1.0)
Coarctation of the aorta	4 (0.9)	1 (1.8)	5 (1.0)
Patent ductus arteriosus	4 (0.9)	-	4 (0.8)
Atrioventricular septal defect	4 (0.9)	-	4 (0.8)
Tricuspid atresia	2 (0.4)	-	2 (0.4)
Transposition of great vessels^†^	1 (0.2)	-	1 (0.2)
Double outlet right ventricle^†^	1 (0.2)	-	1 (0.2)
Ebstein anomaly	-	1 (1.8)	1 (0.2)
Digestive	24 (5.4)	4 (7.2)	28 (5.6)
Esophageal atresia	13 (2.9)	1 (1.8)	14 (2.8)
Anal atresia	10 (2.2)	1 (1.8)	11 (2.2)
Congenital absence, atresia and stenosis of the small intestine	1 (0.2)	2 (3.6)^‡^	3 (0.6)
Genitourinary	13 (2.9)	2 (3.6)	15 (3.0)
Hypospadias	10 (2.2)	-	10 (2.0)
Indeterminate sex	2 (0.4)	-	2 (0.4)
Renal agenesis, bilateral^‡^	-	2 (3.6)	2 (0.4)
Bladder exstrophy^†^	1 (0.2)	-	1 (0.2)
Musculoskeletal	51 (11.5)	1 (1.8)	52 (10.4)
Talipes equinovarus	33 (7.4)^‡a^	-	33 (6.6)
Polydactyly	8 (1.8)	-	8 (1.6)
Syndactyly	5 (1.1)	-	5 (1.0)
Limb reduction	3 (0.6)	-	3 (0.6)
Arthrogryposis^‡^	1 (0.2)	1 (1.8)	2 (0.4)
Craniosynostosis	1 (0.2)	-	1 (0.2)
Abdominal	20 (4.5)	3 (5.4)	23 (4.6)
Gastroschisis	9 (2.0)	2 (3.6)^‡^	11 (2.2)
Diaphragmatic hernia	6 (1.3)	-	6 (1.2)
Omphalocele	5 (1.1)	1 (1.8)	6 (1.2)
Other/multiple	36 (8.1)	17 (30.9)	53 (10.6)
Goldenhar/OAVS^§ ^(OMIM 164210)	9 (2.0)	2 (3.6)	11 (2.2)
VATER/VACTERL^§ ^(OMIM 192350)	7 (1.5)	1 (1.8)	8 (1.6)
Pierre-Robin^‡ ^(OMIM 261800)	5 (1.1)	-	5 (1.0)
MURCS^§^ (OMIM 601076)	1 (0.2)	-	1 (0.2)
OEIS^§^ (OMIM 258040)	1 (0.2)	-	1 (0.2)
Prune belly^‡^ (OMIM 100100)	1 (0.2)	-	1 (0.2)
Sturge-Weber^‡^ (OMIM 185300)	1 (0.2)	-	1 (0.2)
KTW syndrome (OMIM 149000)	1 (0.2)	-	1 (0.2)
CMN syndrome (OMIM 271520)	-	1 (1.8)	1 (0.2)
Congenital ichthyosis	1 (0.2)	-	1 (0.2)
Situs inversus^†^	1 (0.2)	-	1 (0.2)
Sirenomelia^†^	-	1 (1.8)	1 (0.2)
Multiple congenital malformations, not classified	8 (1.8)	12 (21.8)	20 (4.0)
